# Genome-wide identification and expression analysis of the CBF transcription factor family in *Lolium perenne* under abiotic stress

**DOI:** 10.1080/15592324.2022.2086733

**Published:** 2022-06-17

**Authors:** Dan Wang, Binyu Cui, Hanyu Guo, Yaxi Liu, Shuming Nie

**Affiliations:** Key Laboratory of Southwest China Wildlife Resources Conservation (Ministry of Education), College of Life Science, China West Normal University, Nanchong, Sichuan, China

**Keywords:** LpCBF, *perennial ryegrass*, expression level, abiotic stress, cold tolerance

## Abstract

C-repeat binding factor (CBF) subfamily genes encoding transcriptional activators are members of the AP2/ERF superfamily. CBFs play important roles in plant tolerance to abiotic stress. In this study, we identified and analyzed the structure, phylogeny, conserved motifs, and expression profiles of 12 CBFs of the grass species *Lolium perenne* cultured under abiotic stress. The identified LpCBFs were grouped into three phylogenetic clades according to their protein structures and motif organizations. *LpCBF* expression was differentially induced by cold, heat, water deficit, salinity, and abscisic acid, among which cold treatment induced *LpCBF* gene expression significantly. Furthermore, association network analysis indicated that different proteins, including certain stress-related proteins, potentially interact with LpCBFs. Altogether, these findings will enhance our understanding of LpCBFs protein structure and function in the regulation of *L. perenne* stress responses. Our results will provide valuable information for further functional research of LpCBF proteins in *L. perenne* stress resistance.

## Introduction

C-repeat binding factor (CBF) proteins comprise a subfamily of the AP2 transcription factor superfamily. It has been established that these proteins play important roles in facilitating resistance to abiotic stress, particularly low-temperature stress, thus enhancing cold resistance.^1^ For example, it has been demonstrated that CBF proteins can bind specifically to dehydration-responsive element/C-repeat *cis*-acting elements in downstream gene promoters,^2^ thereby regulating the transcription of downstream *COR* (cold-regulated) genes in response to low temperatures.

Previously, *CBF* subfamily genes have been isolated, identified, and further studied in the model plant *Arabidopsis*.^3^ Subsequently, *CBF* genes homologous to those in *Arabidopsis* were identified in numerous species, including *Solanum Lycopersicum*,^4^ *Oryza sativa L*.,^5^ *Prunus persica*,^6^ *Populus hopeiensis*,^7^ and *Kandelia candel*,^8^ thereby confirming that these are common genes among plants. Furthermore, it has been demonstrated that *CBF* family genes are induced by a range of different abiotic stress conditions, with the characteristics and levels of expression differing in each case. For example, expression of an *LlCBF genes* from *Lepidium latifolium* was found to be enhanced in response to high salinity, dehydration, and low-temperature treatments.^9^ Similarly, among the six *CBF* subfamily genes from *P. persi*ca (*PpCBF1-6*), the expression of *PpCBF1*, −5, *and −6* was observed to be induced by exposure to low temperature, whereas that of the other three *PpCBF* genes remained relatively constant.^10^

As key transcription factors that respond to several abiotic stress factors, *CBF* genes can also activate the expression of other stress responsive genes,^11^ as indicated by RNA-Seq analysis of *Arabidopsis* mutants, which revealed that 134 genes are regulated by *CBF* genes.^12^ Furthermore, CBFs reportedly play an important role in the regulation of low-temperature responses in higher plants.^13^ For example, *CBFs* have been shown to upregulate the expression of *COR* genes that promote increased accumulation of proline and soluble sugars, thereby enhancing plant resistance to low temperature.^14^ Similarly, *Phalaenopsis aphrodite PaCBF1* was cold-induced, promoting the upregulated expression of cold-regulated genes *COR6.6* and *RD29a*, thereby protecting plants from cold damage.^15^ Furthermore, Lee found that the expression of eight *CBF/DREB1* genes in *Brassica rapa* increased during cold (4°C) treatment.^16^ Consistently, Barrero-Gil showed that overexpression of *AtCBF3* increased tolerance to freezing stress in *Arabidopsis*,^17^ with cold resistance being enhanced by an accumulation of *CBF* transcripts in response to cold treatment duration. Similarly, Zarka found that the transcript levels of *AtCBF1, AtCBF2*, and *AtCBF3* were enhanced and reached relatively high levels at approximately 3 h after transferring *Arabidopsis* plants from 20°C to 4°C.^18^ Additionally, enhancement of the transcriptional levels of *SlCBF1* and *SlCBF2* has been observed in tomato after exposing plants to a temperature of 10°C,^19^ and ectopic expression of *Lolium perenne LpCBF3* has been found to enhance the cold resistance of transgenic *Arabidopsis* plants.^20^ However, the function of *LpCBFs* needs to be further studied to enable enhancement of the increased reversibility of *L. perenne* plants.

The grass species *L. perenne* adapts to a wide range of soils and is used extensively in animal husbandry as livestock forage, and as a garden ground cover plant.^21^ Accordingly, as a good type of lawn and forage grass, genome-wide analysis of *L. perenne CBF* genes is of particular importance with respect to breeding of varieties resistant to abiotic stress. In this study, we examined 12 *LpCBF* genes obtained from the NCBI database. On the basis of BLAST analysis of multiple sequences, these 12 *CBF* genes were clustered into three groups. Additionally, we searched for gene sequences homologous to *CBF* in other species that may have similar evolutionary histories and functions, and subjected these to phylogenetic analysis. *CBF* expression analyses were conducted on seedlings exposed to a range of abiotic stress conditions, namely high temperature, low temperature, water deficit, salinity, and abscisic acid (ABA) stress. Our findings enabled us to establish the basic characteristics of the CBF subfamily in *L. perenne* and have practical applications in molecular breeding programs. Furthermore, our data provide a useful reference for further studies on CBF mechanisms of action and have practical applications in molecular breeding.

## Materials and methods

### Database search for CBF protein sequences

Sequences of *LpCBF* subfamily genes were retrieved from the NCBI online (https://www.ncbi.nlm.nih.gov/) with *Lolium perenne* (taxid: 4522) database, and based on the findings of previous studies.^22^ LcCBF sequences were further authenticated based on the conserved domains using CDD (http://www.ncbi.nlm.nih.gov/Structure/cdd/wrpsb.cgi) and SMART (http://smart.embl-heidelberg.de/). *Arabidopsis* subfamily *AtCBF* genes were retrieved from the TAIR database (https://www.arabidopsis.org/) with reference to the study by Hu et al.^23^ The protein sequences of CBFs from other gramineous species were retrieved from the NCBI database, and with reference to previous studies, including those on *Oryza sativa*,^24^ *Triticum aestivum*,^25^ *Secale cereale*,^26^ and *Hordeum vulgare*.^27^ The structural characteristics and isoelectric point (pI), molecular weight (MW), and grand average of hydropathicity (GRAVY) values of LpCBF proteins were determined using the ExPASy ProtParam tool (http://web.expasy.org/protparam/), while the subcellular localization of *LpCBF* genes was predicted using UniProt (https://www.uniprot.org/) and WoLF PSORT (https://wolfpsort.hgc.jp/) online tools.

## Sequence alignment and phylogenetic analysis

In order to examine the evolutionary relationship among CBF proteins, multiple amino acid sequences were aligned using Clustal 2.1 software with default settings. An unrooted phylogenetic tree was constructed using MEGA7.0, based on the neighbor-joining (NJ) method and a Poisson substitution model with 1000 bootstrap replications was calculated.

## Structural analysis of LpCBF proteins

The motifs of assessed LpCBF protein*s* were identified using MEME online software (https://meme-suite.org/meme/tools/meme) with advanced default settings. Details of the top 10 predicted motifs were obtained from the MEME suite. The conserved domains of LpCBF proteins were predicted using the SMART web server tool (http://smart.embl-heidelberg.de/), and the three-dimensional structure of LpCBF proteins was predicted using the SWISS-MODEL online server (https://swissmodel.expasy.org/) with advanced default settings. A three-dimensional template for modeling LpCBF proteins was determined based on the order of GMQE.

## Plant growth conditions and stress treatments

For the purposes of the present study, the Yatsyn ecotype of *L. perenne* was used as an experimental material. After sterilization with 1% sodium hypochlorite, selected fully mature seeds were planted in pots containing perlite and thereafter incubated in a growth chamber at 24°C and 65% relative humidity under a 16 h/8 h (day/night) photoperiod. Thirty-day-old seedlings were used in stress treatments. The seedlings were placed in an incubator at 4°C and 42°C for the low- and high-temperature treatments, respectively. For PEG treatment, we removed the original culture solution and then watered the solution with 15% PEG. For the salt treatment, the original culture solution was removed and the solution was watered with 250 mM NaCl. In turn, the water was replaced with a 100 μΜ ABA solution for the ABA treatment. Leaf-blade tissue samples were collected for analysis from each of the five treatments after 0, 3, 6, 12, and 24 h of exposure to the various stress conditions. In each treatment, 20 plants and five plant leaves were collected from different seedlings per treatment.

## Gene expression analysis

Total RNA was extracted from the leaves of *L. perenne* seedlings using plant isolation kits (Sangon Biotech, Shanghai, China, Cat. #B518631). Complementary DNA (cDNA) was prepared from the isolated total RNA using a cDNA synthesis kit with random primers (Vazyme, Nanjing, China, Cat. #R312-02). The cDNA thus obtained was used as a template for quantitative real-time PCR (qPCR) analysis using a SYBR Green real-time PCR master mix (Vazyme, Nanjing, China, Cat. #Q711-02); qRT-PCR was conducted in a CFX96 real-time system (Bio-Rad, Hercules, CA, USA) following the manufacturer’s instructions. Primers are designed to exclude conservative sequences. The primers used for qRT-PCR amplification are listed in Table S1. *L. perenne eIF4A* (eukaryotic translation initiation factor 4 alpha) was used as a reference gene. Gene expression in response to different stress treatments was analyzed over time relative to that recorded at 0 h, with relative gene expression being quantified using the 2^−ΔΔCT^ method. PCR was performed in a 10 μL amplification volume consisting of 5 μL of 2X SYBR Green Mix, 0.2 μL of forward and reverse primer (10 μM), 2 μL of cDNA (50 ng/μL), and 2.6 μL of ddH_2_O. The PCR program was as follows: 1 cycle of 95°C for 30 s, followed by 40 cycles of 95°C for 10 s, 60°C for 30 s, and 72°C for 30 s. The primers used are shown in Table S1, and the specificity of primer pairs was evaluated by dissociation-curve analysis. The resulting clusters were visualized with MeV software.

## Prediction of interacting proteins

To identify candidate proteins that interact with LpCBFs, we analyzed the protein interaction network of LpCBF and used the STRING software (https://string-db.org/cgi/input.pl) to predict the interacting proteins, with *Triticum aestivum* as the background and advanced default settings. Functional enrichment in the network was determined based on local network clusters (STRING).

## Statistical analysis

All the data in this study were analyzed using SPSS version 17.0 and the least significant difference (LSD) test. The means and standard errors were calculated, and p < .05 was considered statistically significant in different gene expressions.

## Results

### *Identification and characterization of* CBF *genes in* L. perenne

We identified 12 *L. perenne CBF* genes in the NCBI GenBank database and predicted their physicochemical characteristics (Table 1). The corresponding LpCBFs-encoded proteins had lengths ranging from 210 to 252 amino acid residues, molecular weights between 22.53 and 26.30 kDa, and isoelectric points ranging from 4.81 to 7.10. The overall average hydropathicity GRAVY values of all predicted LpCBF proteins were negative, ranging from −0.541 to −0.125, thereby implying their hydrophilic nature. The subcellular localization of the LpCBF proteins was examined using UniProt software and predicted using WoLF PSORT (Table S2). The detection of nuclear localization signal (NLS) sequences indicated that these proteins were localized in the cell nucleus.

**Table 1.** Protein physical and chemical properties of *LpCBF* gene family in *Lolium perenne.*

Gene name		Accession/locus ID	CDS (bp)			Protein length (aa)	MW (KD)	pI		GRAVY	Subcellular localization
*LpCBF3a*	ABK32847			714	237		25.52		4.91	−0.468	Nucleus
*LpCBF3b*	AAX57275			672	223		24.46		4.81	−0.437	Nucleus
*LpCBF3c*	ABK32848			714	237		25.55		4.84	−0.453	Nucleus
*LpCBFVb*	BAF36846			630	210		22.53		7.06	−0.145	Nucleus
*LpCBFIVb*	BAF36844			675	224		24.32		7.10	−0.456	Nucleus
*LpCBFIVa*	BAF36843			672	223		24.51		6.97	−0.541	Nucleus
*LpCBFIIIc*	BAF36842			744	247		25.76		5.68	−0.331	Nucleus
*LpCBFIIIb*	BAF36841			759	252		26.30		5.12	−0.327	Nucleus
*LpCBFIIIa*	BAF36840			726	242		25.24		5.23	−0.323	Nucleus
*LpCBFII*	BAF36839			711	236		25.21		5.27	−0.125	Nucleus
*LpCBFIb*	BAF36838			*729*	*242*		*25.74*		*4.90*	*−0.303*	*Nucleus*
*LpCBFIa*	BAF36837			*714*	*237*		*24.99*		*4.84*	*−0.190*	*Nucleus*

Note: CDS, coding sequence length of LpCBF genes; MW, molecular weight of LpCBF proteins; pI, isoelectric point; GRAVY, grand average of hydropathicity.

## Phylogenetic analysis

**Figure 1.**
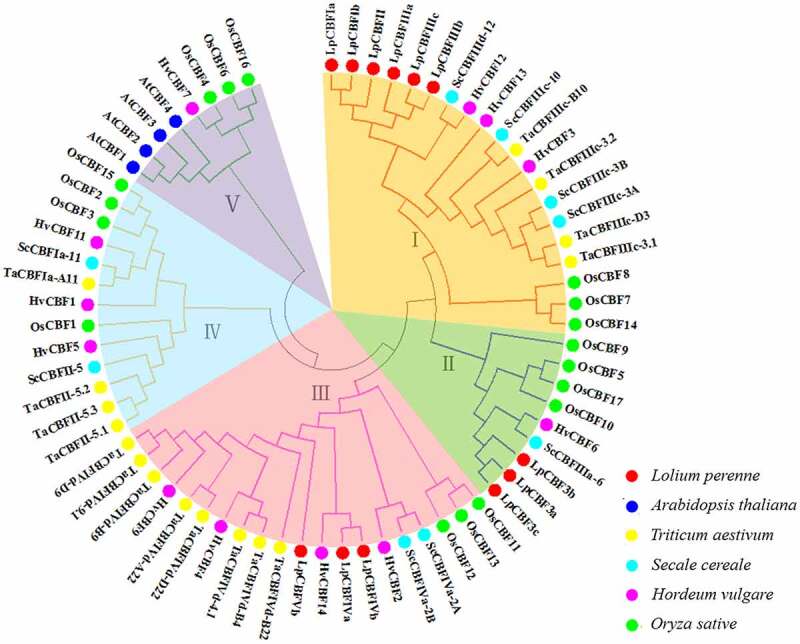
Phylogenetic relationships among CBF proteins of different plants species. The relationships among CBF proteins were inferred from a phylogenetic tree constructed using the neighbor-joining method. The numbers shown at tree nodes indicate the percentage bootstrap replicate trees in which the associated taxa clustered together. Labels above nodes indicate the source species of the respective CBF proteins.

To examine the evolutionary relationships of CBFs found in *L. perenne* with those of other plant species, we constructed an unrooted phylogenetic tree comprising 70 CBF protein sequences from different species, namely, the model plant *Arabidopsis*, and the gramineous species, *L. perenne, O. sativa, T. aestivum, H. vulgare*, and *S. cereale* (Figure 1, Table S3). CBF proteins from the above species were divided into five groups. In addition, proteins LpCBFIIIc, LpCBFIIIb, LpCBFIIIa, LpCBFII, LpCBFIb, and LpCBFIa were found to be highly similar, clustering on the same branch, indicating that these six proteins may have maintained conserved structural fragments during the course of evolution. Further, LpCBF3a, LpCBF3b, and LpCBF3c apparently bear relatively close genetic relationships with HvCBF6 and ScCBFIIIa-6, and were clustered together with certain proteins from *T. aestivum* in common composition group II. LpCBFIVa and LpCBFIVb were clustered as an orthologous pair with HvCBF14 grouped at the secondary end of a clade, suggesting a close genetic relationship, whereas LpCBFVb was located on a different short branch of group III. The tree indicated that none of the assessed *L. perenne* CBFs clustered in either group IV or V, the former of which contained all assessed *CBF* genes from *Arabidopsis* (Figure 1). It should be noted that our assessment of genetic relationships among *CBF* genes was determined by the branch length of the phylogenetic tree, which was constructed based on sequence alignment.

## Multiple sequence alignments

**Figure 2.**
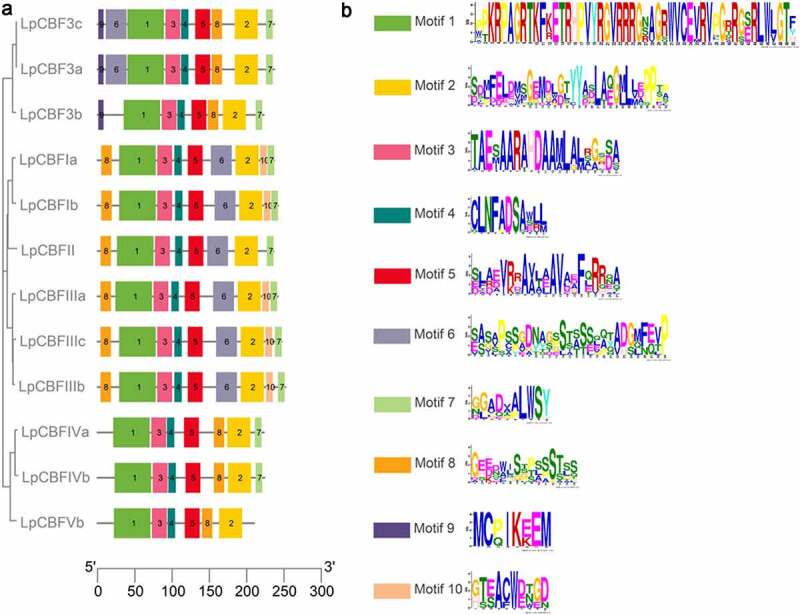
Different motifs analysis diagram in LpCBF proteins. (a) Different motifs were identified using the MEME search tool. Different motifs are indicated by different colors. Boxes represent conserved motifs among the proteins analyzed. (b) Amino acid chain-length and sequence information for Motif 1 to 10.

The multiple sequence alignments of the *LpCBF* genes were determined based on the protein sequence, which enabled us to assign the 12 *LpCBF* genes into three groups (Figure 2a). Furthermore, LpCBF protein sequences were compared to assess amino acid homology, and possible duplication mechanisms were assessed using a matrix of protein sequence identities/positives and sequence coverage (Table S4). Predictably, we obtained high scores for identities and positives among members of the same group.

Further, we used the MEME program to identify common motifs in LpCBF proteins, the 10 most highly enriched of which are shown in Figure 2a. The location and number of motifs were found to be very similar among the members clustering on the same branch of the phylogenetic tree, such as LpCBF3a, LpCBF3b, and LpCBF3c, with LpCBF3a and LpCBF3c being located on the same short branch and characterized by almost identical motif patterns. Nine motifs were identified in CBFs located on the second largest branch, except for LpCBFII, which lacked motif 10. A similar pattern was detected for CBFs on the third branch. Among these motifs, further analysis of motif 1 revealed an AP2 domain (50 amino acid residues), a common feature of the CBF family. The sequence and structural characteristics of other motifs (motifs 2–10) are shown in (Figure 2b). Multiple sequence alignments of the CBF gene family revealed that similar motif structures were clustered together on a single branch, implying their functional similarity.

## Conserved domain analysis and three-dimensional model prediction

**Figure 3.**
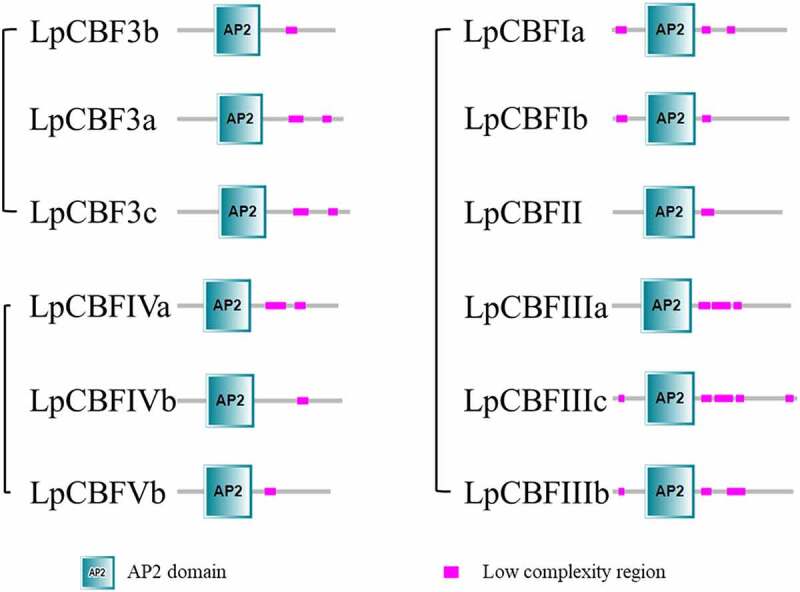
Analysis of LpCBF-protein conserved domains. The conserved domains of LpCBF proteins were identified using SMART software, and the different domains are indicated by different colors.

Multiple sequence analysis showed that the LpCBF protein contained an AP2 domain, a PKKPAGR motif (PKKPAGRxKFxETRHP) and a conserved sequence of DSAWR (Figure S1). Using the SMART program to investigate the conserved domains within LpCBF protein sequences (Figure 3), a very similar distribution of conserved domains among all LpCBF families was revealed, as they were all found to contain an AP2 DNA-binding domain that has been shown to play an important role in biotic responses.

**Figure 4.**
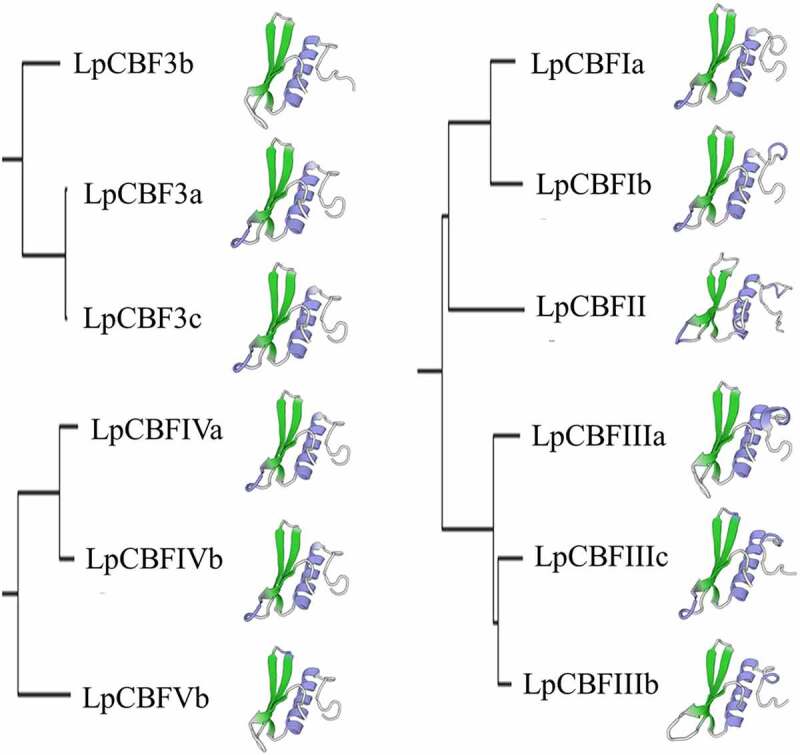
The three-dimensional structures predicted using the Swiss-MODEL server was shown for each of the analyzed proteins in the LpCBF-protein subfamily. Sequence identities >30% between the template and targets were obtained. Template model selection was based on the model with the highest GMQE score.

The higher-level structure of plant proteins is related to their biological function and activity. In this study, we examined the three-dimensional structure of LpCBF proteins based on predictive modeling. Accordingly, we found that the LpCBF family members are highly similar with respect to α-helix and β-sheet configurations, with only certain small differences in random coils being identified (Figure 4). LpCBF sequence identity was found to range from 46.03% to 52.46%, and the GMQE and QMEAN trends were also small (Table S5). The template used to generate the three-dimensional model of LpCBF proteins was measured relative to the structure of the complex of GCC-box binding domain by solution nuclear magnetic resonance spectroscopy. Therefore, the three-dimensional structure of LpCBFs may play an important role in transcriptional regulation.

## *LpCBF* gene expression in response to abiotic stress

**Figure 5.**
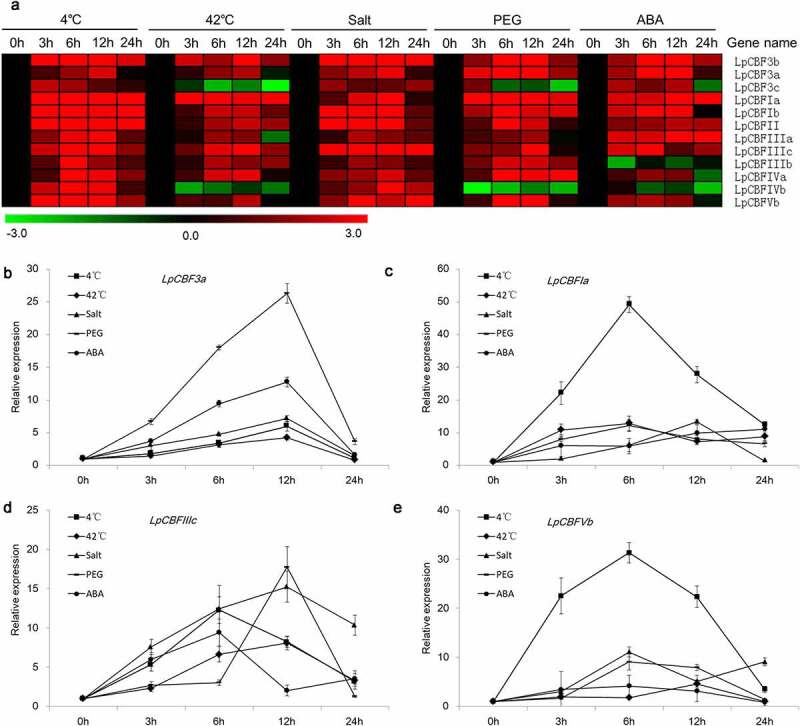
Temporal expression of 12 *LpCBF* genes in response to diverse stress factors. Heat map of the 12 *LpCBF* genes in seedlings subjected to 4°C, 42°C, salinity, water deficit, and ABA stress treatments over a 12-h time course (a). Temporal expression of *LpCBF3a* (b), *LpCBFIa* (c), *LpCBFIIIc* (d) and *LpCBFVb* (e) under 4°C, 42°C, salinity, PEG 6000 and ABA stress treatments.

To identify the functional role of *CBFs* in *L. perenne*, we used qRT-PCR to analyze gene expression in response to the exposure to different abiotic stress factors including cold, heat, salinity, PEG 6000, and ABA stress. Our results showed that the 12 *CBF* genes under study responded to different extents to the abiotic stress conditions tested (Figure 5a, Table S7). Most of the 12 genes were upregulated across the stress treatments, with all 12 *CBFs* being upregulated in response to cold (4°C). We observed that when the cold stress duration was extended, the expression of the 12 *CBF* genes initially increased and subsequently decreased with time, generally peaking after 6 h under stress. The only exception in this regard was *LpCBF3a*, the expression of which peaked at 12 h. The expression of 10 of the *CBF* genes was also upregulated under 42°C and water deficit treatments, whereas that of the remaining two (*LpCBF3c* and *LpCBFIVb*) was down-regulated under the same conditions. Meanwhile, the expression of nine *CBFs* was markedly upregulated under salinity stress, whereas that of *LpCBF3c, LpCBFIIIa*, and *LpCBFIIIb* was slightly upregulated when exposed to high salinity. Ten *CBFs* also showed upregulated expression in response to ABA stress, whereas the expression of the remaining two (*LpCBFIIIb and LpCBFIVb*) was down-regulated (Figure 5a).

The expression of four genes, namely, *LpCBF3a, LpCBFIa, LpCBFIIIc*, and *LpCBFVb*, was found to be markedly upregulated in response to all five stress treatments, particularly in seedlings exposed to 4°C; furthermore, among these genes, the expression of *LpCBFIa* and *LpCBFIVb* was markedly higher under the 4°C treatment than under any other stress condition (Figure 5b-e). Additionally, we compared the expression of the 12 *CBFs* in seedlings that had been exposed to 4°C for 6 and 12 h. In this case, we found that, at both 6 and 12 h, the expression of *LpCBFIa* was highest and that the expression of *LpCBF3b, LpCBFIb*, and *LpCBFVb* was higher than that of other genes at 6 h, whereas the expression of *LpCBF3b, LpCBFIb, LpCBFII, LpCBFIIIa*, and *LpCBFVb* was higher than that of other genes when exposed to 4°C for 12 h (Figure S2). Furthermore, we established that the response of these genes to cold stress was treatment duration-dependent. These findings indicated that *LpCBFs* may potentially play an important role in cold resistance in *L. perenne*, particularly, *LpCBF3a, LpCBFIa, LpCBFIIIc*, and *LpCBFVb.*

## Prediction of interacting proteins

**Figure 6.**
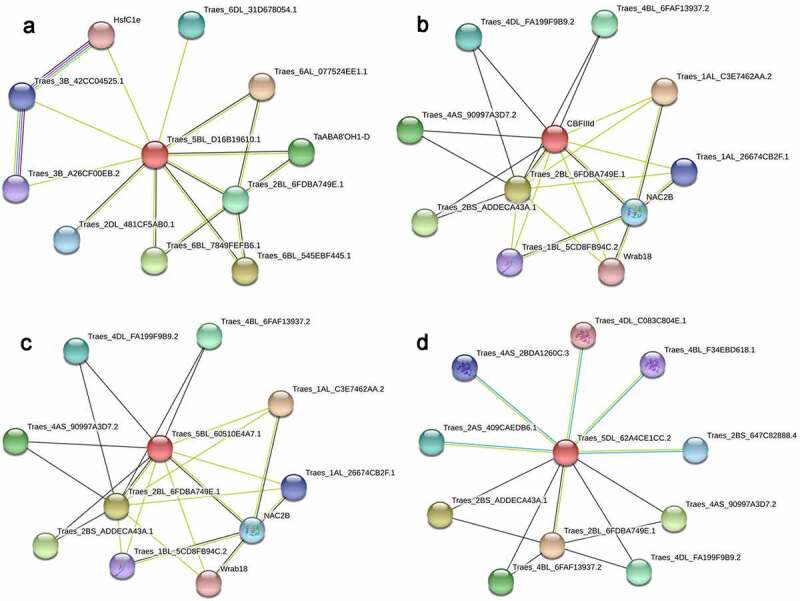
Predicted protein interaction networks of LpCBF3a (a), LpCBFIa (b), LpCBFIIIc (c), and LpCBFVb (d). Protein interaction networks were generated using STRING online with *Triticum aestivum* as the background. Edges represent protein–protein associations, nodes represent proteins, and red nodes represent query proteins. Black, green, blue, light sky-blue, and purple lines represent co-expression, text-mining, gene co-occurrence, protein homology, and experimental determination, respectively.

To gain a further insight into the function of LpCBF proteins, we sought to predict the proteins with which LpCBFs might interact. To this end, we generated protein association networks for LpCBF3a, LpCBFIa, LpCBFIIIc, and LpCBFVb (Figure 6a-d). A number of different proteins were predicted to interact with these four LpCBF proteins, thereby suggesting a regulatory function. Somewhat surprisingly, different models were predicted for LpCBFIa and LpCBFIIIc, although the associated proteins were the same, thereby indicating that they play similar functional roles (Figure 6b, c). Two of the predicted proteins, NAC2b and LEA (late embryogenesis abundant), are known to be associated with stress. NAC transcription factors have been shown to be involved in plant tolerance to abiotic stress,^28^ a member of the LEA protein family, which is abundant in seeds and pollen, and plays a regulatory role in tolerance against multiple abiotic stress conditions.^29^ We also predicted LEA proteins that interact with LpCBF proteins in the network based on functional enrichments (Table S6). Overall, these findings indicate that LpCBF proteins play regulatory roles in response to adverse environmental stimuli, although the underlying mechanisms may differ.

## Discussion

*CBF* gene families have been identified in a number of plant species,^23^ and sequences have also been analyzed in rye (*Secale cereal*),^30^ wheat (*Triticum aestivum*),^31^ cotton (*Gossypium hirsutum*),^32^ and barley (*Hordeum Vulgare*).^33^ In this study, we identified 12 *CBF* genes in the grass species *Lolium perenne*, which were classified into three groups (Table 1). Phylogenetic analysis revealed that among the species under study, LpCBF sequences are more closely related to those of *T. aestivum, S. cereale*, and *H. vulgare* than to those of *O. sativa* (Figure 1). Although the sequences tend to be highly conserved and are predicted to have a similar function, it has been found that different *CBF* family genes within the same species can differ with respect to structure and function, despite having similar sequences. Genome-wide identification and analysis are often used to study gene families;^34^ further, bioinformatics-based control of protein function is a practical method in gene research.^35^ To gain further understanding of the properties of LpCBF proteins, we performed conserved domain and three-dimensional modeling analyses, the results of which revealed that LpCBF family members typically have conserved domains and protein structures (Figure 3 and 4). Furthermore, our comparative genome sequence analysis using the MEME program indicated that LpCBF members within the same clade are characterized by the same motifs (Figure 2). The diversity of CBF sequences in terms of phylogenetic relationships, structural and functional characteristics, and regulatory levels contribute to the differentiation in the regulatory properties of these proteins.^36^

The functions of some *CBF* transcription factor genes have been determined based on annotation analysis that has established that *CBF* genes play roles in the response of plants to different abiotic stress factors.^37^ Moreover, examination of the different expression patterns of orthologous *CBF* genes under different stress conditions suggests that these genes may be involved in different regulatory pathways.^38^ Similarly, our analysis of the expression profiles of *LpCBF*s reported herein revealed differential expression in response to low/high temperature, water deficit, salinity, and ABA treatments (Figure 5). We found that the expression of all 12 *LpCBFs* studied was upregulated in seedlings exposed to cold (4°C), initially increasing and subsequently decreasing when the stress period was extended (Figure 5). These observations are consistent with those reported by Han,^39^ who demonstrated that cold treatment (4°C) induced the expression of *LpCBF3*. Similarly, our findings are consistent with those obtained for CBF family members in other plants. For example, among the CBF homologs *LeCBF1–3* in tomato (*Lycopersicon esculentum*), only the *LeCBF1* gene was found to be cold-inducible.^40^ Similarly, in *Arabidopsis, AtCBF1, AtCBF2*, and *AtCBF3* were shown to be induced in response to low temperature,^11^ and in papaya (*Carica papaya*), the expression of *CBF1* and *CBF2* was observed to increase under low temperature.^41^ Furthermore, the findings of Zhao regarding low temperature-induced sensitivity and resistance of double mutants indicated that *AtCBF2* plays a more important role than either *AtCBF1* or *AtCBF3* in freezing tolerance, whereas in *Malus sieversii* observed different expression patterns for four *CBF* homolog genes in response to a period of cold treatment.^42,43^ Altogether, these findings provide important evidence indicating that *LpCBFs* play an important role in the resistance to low-temperature stress.

Previous studies have indicated that in addition to low temperature, the expression of *CBFs* is similarly induced by other stress factors. For example, Jiang found that the expression of *CBF* homologs was induced by exposure to low temperature, salinity, and water deficit stress,^44^ while B yun observed that *DaCBF7* was induced by water deficit, cold, and salinity.^45^ In turn, Li demonstrated that the *MtCBF4* gene from *Medicago truncatula* plays an important role in responses to water deficit and salinity stress conditions.^46^ Consistently, herein, we found that the expression of most of the 12 *LpCBFs* studied was also induced under salinity and water deficit (Figure 5a). Studies have also revealed that ABA treatment induces the expression of CBF homologs, thereby indicating that an ABA-inducible signaling pathway is involved in the regulation of cold-regulated genes via the CRT promoter element.^47^ Consistent with this assumption, the exogenous application of ABA has been shown to enhance the expression of *VvCBF2, VvCBF3, VvCBF4*, and *VvCBF6*,^48^ and Peng observed that, whereas the *BgCBF1* gene showed limited induction in response to salinity and water deficit stress, it exhibited relatively high upregulated expression following ABA treatment.^49^ In this regard, it has been reported that in the regulation of OST1 (open stomata 1), a Ser/Thr protein kinase in the ABA signaling pathway interacts with ICE1, an upstream regulator involved in the CBF pathway.^50^ Here, we found that the expression of 10 of the 12 assessed *LpCBFs* was up-regulated in response to ABA treatment, whereas that of the remaining two *LpCBFs* was down-regulated (Figure 5a). These observations indicate a complex and intimate interaction between the ABA and the CBF pathways.

One of the primary objectives of this study was to determine whether candidate *LpCBFs* play a role in plant adaptation. On the basis of our analysis of expression profiles, we identified four candidate genes (*LpCBF3a, LpCBFIa, LpCBFIIIc*, and *LpCBFVb*) that were highly expressed in response to exposure to multiple stress factors (Figure 5b-e). Notably, these four candidate genes were induced in seedlings subjected to low temperature (4°C), water deficit, and salinity stress; therefore, we speculate that they may play regulatory roles in the stress responses of *L. perenne*, which warrants further examination (Figure 5b-e). These results are also consistent with those reported previously, indicating that the *SpCBF1* gene from *Solanum pinnatisectum*, which is induced by chilling stress, plays an important role in chilling stress.^51^ CBFs have been shown to participate in cold-induced activation of the transcriptional response,^52^ and the CBF subfamily has been found to be dependent on a low-temperature signal chilling pathway.^53^ In addition, *CBFs* have been demonstrated to affect plant growth and development,^54^ while *PpCBF1* has been shown to be involved in regulation of the bud endodormancy process in pears.^35^ Furthermore, CBFs have been established to play regulatory roles in the tolerance to metal stress, such as that induced by cadmium and molybdenum treatment.^55^ Altogether, these findings imply that CBF members play distinct roles in the response to abiotic stress, whereby, it would be desirable to further investigate the functions of the *LpCBF* genes identified in the present study.

*CBFs* have previously been characterized as transcriptional factors that contribute to transcriptional regulation in response to abiotic stress;^56^ furthermore, cold response-related genes implicated in the response to low temperature have been identified and the underlying mechanisms characterized.^57^ Here, we used the aforementioned candidate genes as a basis to predict LpCBF protein association networks (Figure 6 and Table S6). Our data revealed that these CBF proteins potentially interact with NAC transcription factors and LEA proteins, as well as other currently uncharacterized proteins which, accordingly, warrant further study to enable a functional characterization of their roles in *L. perenne*. Similarly, further in-depth research is required to elucidate the mechanisms underlying transcriptional regulatory mechanisms of LpCBFs. Low-temperature environments induce *CBF* genes via associated signal transduction pathways, thereby initiating the subsequent regulation of target genes that mediate the response to low-temperature stress.^58^ For example, it has been demonstrated that MdBBX37 interacts with MdICE1 to enhance the transcriptional activity of MdICE1 in the MdCBF1 pathway, which in turn initiates the regulation of cold stress tolerance-signaling.^59^ Furthermore, CBFs have been demonstrated to enhance cold adaptability by regulating the expression of *COR* genes.^60^

Abiotic stress limits plant growth and development. To counter the resulting adverse effects, plants have evolved sophisticated adaptive capacities to deal with different types of stress.^61^ Furthermore, from the perspective of the application of CBFs to molecular breeding, ectopic expression of *CBF* subfamily genes has been shown to enhance plant tolerance to abiotic stress. For example, the ectopic expression of *HvCBF7* and *HvCBF9a* in *Arabidopsis* seemingly enhances salinity tolerance,^62^ while the ectopic expression of pear *PpCBF1* confers increased cold hardiness.^63^ Similarly, the ectopic expression of *AtCBF3* in *Solanum tuberosum* enhanced the tolerance of transformed plants to high temperature (40°C) stress.^64^ We anticipate that further research on the *L. perenne CBF* genes identified herein will contribute to enhancing environmental adaptation of this grass, and that a combination of modern molecular biology technology and traditional breeding methods will improve the efficiency with which this objective is achieved.

## Conclusion

We identified 12 *LpCBF* genes in the grass species *Lolium perenne* and performed a comprehensive characterization of these genes with respect to protein features, phylogenetic relationships, motifs, and structural domains. Furthermore, our analysis of the expression profiles of *LpCBF* genes in seedlings exposed to different stress conditions indicated that these genes potentially play a variety of roles in response to cold, heat, salinity, water deficit, and ABA stress. In addition, on the basis of association network analysis, we predicted the identity of putative interacting proteins presumably regulated by LpCBFs. We believe the findings reported herein will provide valuable information for further functional research on the role of LpCBF proteins with respect to plant responses to stress and perspective applications in breeding for stress resistance. Finally, our observations provide a sound theoretical basis for functional studies of the *LpCBF* gene family and contribute to developing a potential strategy for further breeding of *L. perenne*.

## Supplementary Material

Supplemental Material

## Data Availability

All data generated or analyzed during this study were included in this published article and the addition-al files.

## Abbreviations

CBF: C-repeat binding factor
AP2/ERF: APETALA2/Ethylene responsive factor
DRE/CRT: dehydration responsive element/C-repeat
COR: Cold regulated
ABA: Abscisic acid
pI: Isoelectric point
MW: Molecular weight
GRAVY: Grand average hydropathicity
NLS: Nuclear localization signal
cDNA: Complementary DNA
PEG: Polyethylene glycol
eIF4A: Eukaryotic translation initiation factor 4 alpha
LEA: Late embryogenesis abundant
NAC: NAM、ATAF1/2、CUC2.

## References

[1] Shi Y, Ding Y, Yang S. Molecular regulation of CBF signaling in cold acclimation. Trends Plant Sci. 2018;S1360138518300864:1–5. doi:10.1016/j.tplants.2018.04.002. 29735429

[2] Jurczyk B, Rapacz M, Budzisz K, Barcik W, Sasal M. The effects of cold, light and time of day during low-temperature shift on the expression of CBF6, FpCor14b and LOS2 in *Festuca pratensis*. Plant Science. 2012;183:143–12. doi:10.1016/j.plantsci.2011.08.004. 22195587

[3] Stockinger JE, Gilmour JS, Thomashow MF. Arabidopsis thaliana CBF1 encodes an AP2 domain-containing transcriptional activator that binds to the C-repeat/DRE, a cis-acting DNA regulatory element that stimulates transcription in response to low temperature and water deficit. Proceedings of the National Academy of Sciences of the United States of America. 1997;94:1035–1040. http://europepmc.org/backend/ptpmcrender.fcgi?accid=PMC19635&blobtype=pdf 10.1073/pnas.94.3.1035 PMC19635 9023378

[4] Xin Zhang SGF, Cheng H, Lou Y, Rhee SY, Stockinger EJ, Michael F, Thomashow MF. Freezing-sensitive tomato has a functional CBF cold response pathway, but a CBF regulon that differs from that of freezing-tolerant arabidopsis. The Plant J. 2004;39:905–919. 15341633 10.1111/j.1365-313X.2004.02176.x

[5] Ito Y, Katsura K, Maruyama K, Taji T, Yamaguchi-Shinozaki K, Seki M, Shinozaki K, Yamaguchi-Shinozaki K. Functional analysis of rice DREB1/CBF-type transcription factors involved in cold-responsive gene expression in transgenic rice. Plant Cell Physiol. 2006;47(1):141–153. doi:10.1093/pcp/pci230. 16284406

[6] Artlip TS, Wisniewski ME, Norelli JL. Field evaluation of apple overexpressing a peach CBF gene confirms its effect on cold hardiness, dormancy, and growth. Environ Exp Bot. 2014;106:79–86. doi:10.1016/j.envexpbot.2013.12.008.

[7] Wang Z, Liu J, Guo H, He X, Wu W, Du J, Zhang Z, An X. Characterization of two highly similar CBF/DREB1-like genes, PhCBF4a and PhCBF4b, in *Populus hopeiensis*. Plant Physiology andBiochemistry. 2014;83:107–116. doi:10.1016/j.plaphy.2014.07.012. 25128646

[8] Du Z, Li J. Expression, purification and molecular characterization of a novel transcription factor KcCBF3 from kandelia candel. Protein Expr Purif. 2019;153:26–34. doi:10.1016/j.pep.2018.08.006. 30118861

[9] Akhtar M, Jaiswal A, Jaiswal JP, Qureshi MI, Tufchi M, Singh NK. Cloning and characterization of cold, salt and drought inducible C-repeat binding factor gene from a highly cold adapted ecotype of *Lepidium latifolium L*. Physiol Mol Biol Plants. 2013;19(2):221–230. doi:10.1007/s12298-012-0154-2. 24431489 PMC3656188

[10] Li L, Bo Z, Yin XR, Xu CJ, Chen KS, Chen K-S. Differential expression of the CBF gene family during postharvest cold storage and subsequent shelf-life of peach fruit. Plant Molecular Biology Reporter. 2013;31(6):1358–1367. doi:10.1007/s11105-013-0600-5.

[11] Sunchung P, Chin-Mei L, Colleen J, Doherty, Sarah J, Kim Y, Thomashow MF. Gilmour: regulation of the Arabidopsis CBF regulon by a complex low-temperature regulatory network. The Plant J. 2015;82(2):193–207. doi:10.1111/tpj.12796. 25736223

[12] Yuxin Jia YD, Ding Y, Shi Y, Zhang X, Gong Z, Yang S. The cbfs triple mutants reveal the essential functions ofCBF sin cold acclimation and allow the definition of CBF regulons in Arabidopsis. New Phytol. 2016;212(2):345–353. doi:10.1111/nph.14088. 27353960

[13] Zhou M, Chen H, Wei D, Ma H, Lin J. Arabidopsis CBF3 and DELLAs positively regulate each other in response to low temperature. Sci Rep. 2017;7(1):39819. doi:10.1038/srep39819. 28051152 PMC5209670

[14] Thomashow MF. Molecular basis of plant cold acclimation: insights gained from studying the CBF cold response pathway. Plant Physiol. 2010;154(2):571–577. doi:10.1104/pp.110.161794. 20921187 PMC2948992

[15] Peng PH, Lin CH, Tsai HW, Lin TY. Cold response in phalaenopsis aphrodite and characterization of PaCBF1 and PaICE1. Plant Cell Physiol. 2014;55(9):1623. doi:10.1093/pcp/pcu093. 24974386

[16] Lee S-C, Lim M-H, Yu J-G, Park B-S, Yang T-J. Biochemistry. Genome-wide characterization of the CBF/DREB1 gene family in *Brassica rapa*. Plant Physiology and Biochemistry. 2012;61:142–152. doi:10.1016/j.plaphy.2012.09.016. 23148914

[17] Barrero-Gil J, Huertas R, Rambla JL, Granell A, Jjpc S. Environment. Tomato plants increase their tolerance to low temperature in a chilling acclimation process entailing comprehensive transcriptional and metabolic adjustments. Plant Cell Environ. 2016;39(10):2303–2318. doi:10.1111/pce.12799. 27411783

[18] Zarka DG, Vogel JT, Cmfjpp T, Thomashow MF. Cold Induction of arabidopsis CBF genes involves multiple ICE (Inducer of CBF Expression) promoter elements and a cold-regulatory circuit that is desensitized by low temperature. Plant Physiol. 2003;133(2):910–918. doi:10.1104/pp.103.027169. 14500791 PMC219064

[19] J S G, M A S, P M S, D J E, F M T. Overexpression of the arabidopsis CBF3 transcriptional activator mimics multiple biochemical changes associated with cold acclimation. Plant Physiol. 2000;124(4):1854–1865. doi:10.1104/pp.124.4.1854. 11115899 PMC59880

[20] Xiong Y, Fei SZ. Functional and phylogenetic analysis of a DREB/CBF-like gene in perennial ryegrass (*Lolium perenne L*.). Planta. 2006;224(4):878–888. doi:10.1007/s00425-006-0273-5. 16614820

[21] Byrne SL, Nagy I, Pfeifer M, Armstead I, Swain S, Studer B, Mayer K, Campbell JD, Czaban A, Hentrup S. A synteny-based draft genome sequence of the forage grass *Lolium perenne*. Plant J. 2015;84(4):816–826. doi:10.1111/tpj.13037. 26408275

[22] Tamura K, Yamada T. A perennial ryegrass CBF gene cluster is located in a region predicted by conserved synteny between Poaceae species. Theor Appl Genet. 2007;114(2):273–283. doi:10.1007/s00122-006-0430-z. 17075706

[23] Hu Z, Ban Q, Hao J, Zhu X, Cheng Y, Mao J, Lin M, Xia E, Li Y. Genome-wide characterization of the C-repeat binding factor (CBF) gene family involved in the response to abiotic stresses in tea plant (*Camellia sinensis*). Front Plant Sci. 2020;11:921. doi:10.3389/fpls.2020.00921. 32849669 PMC7396485

[24] Cao Y, Wang J, Guo L, Xiao K. Identification, characterization and expression analysis of transcription factor (CBF) genes in rice (*Oryza sativa L*.). Fronters of Agriculture in China. 2008;2(3):253–261. doi:10.1007/s11703-008-0052-0.

[25] Badawi M, Da Nyluk J, Boucho B, Houde M, Sarhan F. The CBF gene family in hexaploid wheat and its relationship to the phylogenetic complexity of cereal CBFs. Molecular Genetics & Genomics. 2007;277(5):533–554. doi:10.1007/s00438-006-0206-9. 17285309 PMC2491707

[26] Campoli C, Matus-Cádiz M, Pozniak CJ, Cattivelli L, Fowler DB. Comparative expression of Cbf genes in the triticeae under different acclimation induction temperatures. Molecular Genetics & Genomics. 2009;282(2):141–152. doi:10.1007/s00438-009-0451-9. 19421778 PMC2757611

[27] Francia E, Morcia C, Pasquariello M, Mazzamurro V, Milc JA, Rizza F, Terzi V, Pecchioni N. Copy number variation at the HvCBF4–HvCBF2 genomic segment is a major component of frost resistance in barley. Plant Molecular Biology. 2016;92(1–2):1–15. doi:10.1007/s11103-016-0505-4. 27338258

[28] Shao HB, Wang HY, Tang XL. NAC transcription factors in plant multiple abiotic stress responses: progress and prospects. Front Plant Sci. 2015;6:902. doi:10.3389/fpls.2015.00902. 26579152 PMC4625045

[29] Zhao X, Zhan L-P, Zou X-Z. Improvement of cold tolerance of the half-high bush Northland blueberry by transformation with the LEA gene from *Tamarix androssowii*. Plant Growth Regul. 2011;63(1):13–22. doi:10.1007/s10725-010-9507-4.

[30] Jung W, Jung Y, Seo W. Identification of novel C-repeat binding factor (CBF) genes in rye (Secale cereale L.) and expression studies. Gene. 2018;684:82–94. doi:10.1016/j.gene.2018.10.055. 30359739

[31] Mohseni S, Che H, Djillali Z, Dumont E, Nankeu J, Danyluk J. Wheat CBF gene family: identification of polymorphisms in the CBF coding sequence. Genome. 2012;55(12):865–881. doi:10.1139/gen-2012-0112. 23231605

[32] Ma LF, Zhang JM, Huang GQ, Li Y, Li XB, Zheng Y. Molecular characterization of cotton C-repeat/dehydration-responsive element binding factor genes that are involved in response to cold stress. Mol Biol Rep. 2014;41(7):4369–4379. doi:10.1007/s11033-014-3308-1. 24566693

[33] Choi DW, Close R, Close TJ. Barley Cbf3 gene identification, expression pattern, and map location. Plant Physiol. 2002;129(4):1781–1787. doi:10.1104/pp.003046. 12177491 PMC166766

[34] Kim Y, Hwang I, Jung HJ, Park JI, Kang JG, Nou IS. Genome-wide classification and abiotic stress-responsive expression profiling of carotenoid oxygenase genes in *brassica rapa* and *brassica oleracea*. J Plant Growth Regul. 2016;35(1):202–214. doi:10.1007/s00344-015-9520-y.

[35] Karanja BK, Xu L, Wang Y, Tang M, M’mbone Muleke E, Dong J, Liu L. Genome-wide characterization of the AP2/ERF gene family in radish (*Raphanus sativus L*.): unveiling evolution and patterns in response to abiotic stresses. Gene. 2019;718:144048. doi:10.1016/j.gene.2019.144048. 31421189

[36] Marozsán-Tóth Z, Vashegyi I, Galiba G, Physiology Btjjo P. The cold response of CBF genes in barley is regulated by distinct signaling mechanisms. Journal of Plant Physiol. 2015;181:42–49. doi:10.1016/j.jplph.2015.04.002. 25974368

[37] Fnjmj S. Arabidopsis CBF1 and CBF3 have a different function than CBF2 in cold acclimation and define different gene classes in the CBF regulon. Proceedings of the National Academy of Sciences of the United States of America. 2007;104(52):21002–21007. http://europepmc.org/backend/ptpmcrender.fcgi?accid=PMC2409256&blobtype=pdf 10.1073/pnas.0705639105 PMC2409256 18093929

[38] Peng YL, Wang YS, Fei J, Sun C. Isolation and expression analysis of two novel C-repeat binding factor (CBF) genes involved in plant growth and abiotic stress response in mangrove *Kandelia obovata*. Ecotoxicology. 2020;29(5–6):718–725. doi:10.1007/s10646-020-02219-y. 32394360

[39] Han Z, Ssjmg B. Genomics. Isolation and characterization of cold-regulated transcriptional activator LpCBF3 gene from perennial ryegrass (*Lolium perenne L*.). Molecular Genetics Genomics. 2008;279(6):585–594. doi:10.1007/s00438-008-0335-4. 18351391

[40] Zhang X, Fowler SG, Cheng H, Lou Y, Thomashow MF, Stockinger EJ, Thomashow MF. Freezing-sensitive tomato has a functional CBF cold response pathway, but a CBF regulon that differs from that of freezing-tolerant Arabidopsis. Plant J. 2010;39:905–919. 10.1111/j.1365-313X.2004.02176.x 15341633

[41] Maurya NK, Goswami AK, Singh SK, Prakash J, Kumari A, Chinnusamy V, Talukdar A, Pradhan S, Kumari A. Studies on expression of CBF1 and CBF2 genes and anti-oxidant enzyme activities in papaya genotypes exposed to low temperature stress. Sci Hortic (Amsterdam). 2019;261:108914. doi:10.1016/j.scienta.2019.108914.

[42] Wang ZH, Tian G, Qin WJ, Qin W, Turdi M. SCI TJEJH. Characterization of CBF1, CBF2, CBF3, and CBF4 genes of Malus sieversii and analysis of their expression in different habitats. Eur J Hortic Sci. 2017;82(2):81–89. doi:10.17660/eJHS.2017/82.2.3.

[43] Zhao C, Zhang Z, Xie S, Si T, Li Y, Jkjpp Z. Mutational evidence for the critical role of CBF transcription factors in cold acclimation in arabidopsis. Plant Physiol. 2016;171(4):2744–2759. doi:10.1104/pp.16.00533. PMC4972280 27252305

[44] Jiang F, Feng W, Zhen W, Ying L, Shi G, Hu J, Hou X. Components of the arabidopsis CBF cold-response pathway are conserved in non-heading Chinese cabbage. Plant Molecular Biology Reporter. 2011;29(3):525–532. doi:10.1007/s11105-010-0256-3.

[45] Byun MY, Lee C, Kang LH, Park TK, Kim SCIWJP, Park H, Lee H, Kim WT. Constitutive expression of DaCBF7, an Antarctic vascular plant *Deschampsia antarctica* CBF homolog, resulted in improved cold tolerance in transgenic rice plants. Plant Sci. 2015;236:61–74. doi:10.1016/j.plantsci.2015.03.020. 26025521

[46] Li D, Zhang Y, Hu X, Shen X, Lei M, Su Z, Wang T, Jjbpb D. Transcriptional profiling of *Medicago truncatula* under salt stress identified a novel CBF transcription factor MtCBF4 that plays an important role in abiotic stress responses. BMC Plant Biol. 2011;11(1):109. doi:10.1186/1471-2229-11-109. 21718548 PMC3146422

[47] Knight H, Zarka DG, Okamoto H, Thomashow MF, Knight MR. Abscisic acid induces CBF gene transcription and subsequent induction of cold-regulated genes via the CRT promoter element. Plant Physiol. 2004;135(3):1710–1717. doi:10.1104/pp.104.043562. 15247382 PMC519084

[48] Rubio S, Noriega X, Pérez FJ. Abscisic acid (ABA) and low temperatures synergistically increase the expression of CBF/DREB1 transcription factors and cold-hardiness in grapevine dormant buds. Ann Bot. 2019;123(4):681–689. doi:10.1093/aob/mcy201. 30418484 PMC6417478

[49] Peng YL, Wang YS, Fei J, Cheng H, Ccje S. Isolation and expression analysis of a CBF transcriptional factor gene from the mangrove *Bruguiera gymnorrhiza*. Ecotoxicology. 29, no. 6 (2020 5):726–735. doi:10.1007/s10646-020-02215-2. 32337665

[50] Ding Y, Hui L, Zhang X, Xie Q, Gong Z, Yang S. OST1 kinase modulates freezing tolerance by enhancing ICE1 stability in arabidopsis. Dev Cell. 2015;32(3):278–289. doi:10.1016/j.devcel.2014.12.023. 25669882

[51] Zhu W, Shi K, Tang R, Mu X, Cai J, Chen M, You X, Yang Q. Isolation and functional characterization of the SpCBF1 gene from *Solanum pinnatisectum*. Physiology and Molecular Biology of Plants. 2018;24(4):605–616. doi:10.1007/s12298-018-0536-1. 30042616 PMC6041227

[52] Guo Y, Xiong L, Ishitani M. An arabidopsis mutation in translation elongation factor 2 causes superinduction of CBF/DREB1 transcription factor genes but blocks the induction of their downstream targets under low temperatures. Proceedings of the National Academy of Sciences of the United States of America. 2002;99(11):7786. https://www.pnas.org/doi/full/10.1073/pnas.112040099 10.1073/pnas.112040099 PMC124352 12032361

[53] Benedict C, Skinner JS, Meng R, Chang Y, Hurry V, Huner NPA, Finn CE, CHEN THH, HURRY V. The CBF1-dependent low temperature signalling pathway, regulon and increase in freeze tolerance are conserved in populus spp. Plant Cell Environ. 2010;29(7):1259–1272. doi:10.1111/j.1365-3040.2006.01505.x. 17080948

[54] Siddiqua M, Nassuth A. Vitis CBF1 and Vitis CBF4 differ in their effect on arabidopsis abiotic stress tolerance, development and gene expression. Plant Cell Environ. 2011;34(8):1345–1359. doi:10.1111/j.1365-3040.2011.02334.x. 21486303

[55] Ali N, Hadi F. CBF/DREB transcription factor genes play role in cadmium tolerance and phytoaccumulation in *Ricinus communis* under molybdenum treatments. Chemosphere. 2018;208:425–432. doi:10.1016/j.chemosphere.2018.05.165. 29885509

[56] Kidokoro S, Watanabe K, Ohori T, Moriwaki T, Yamaguchi Hinozaki K, Mizoi J, Myint Phyu Sin Htwe N, Fujita Y, Sekita S, Shinozaki K. Soybean DREB1/CBF‐type transcription factors function in heat and drought as well as cold stress‐responsive gene expression. Plant J. 2014;81(3):505–518. doi:10.1111/tpj.12746. 25495120

[57] Thomashow FMF, Thomashow MF. Arabidopsis transcriptome profiling indicates that multiple regulatory pathways are activated during cold acclimation in addition to the CBF cold response pathway. Plant Cell. 2002;14(8):1675–1690. doi:10.1105/tpc.003483. 12172015 PMC151458

[58] Novillo F, Medina J, Rodríguez-Franco M, Neuhaus G, Salinas J. Genetic analysis reveals a complex regulatory network modulating CBF gene expression and Arabidopsis response to abiotic stress. J Exp Bot. 2012;63(1):293–304. doi:10.1093/jxb/err279. 21940717 PMC3245470

[59] An X-FW J-P, Zhang X-W, You C-X, Hao Y-J, Hao Y-J. Apple B‐box protein BBX37 regulates jasmonic acid mediated cold tolerance through the JAZ‐BBX37‐ICE1‐CBF pathway and undergoes MIEL1‐mediated ubiquitination and degradation. New Phytol. 2021;229(5):2707–2729. doi:10.1111/nph.17050. 33119890

[60] Liu Y, Dang P, Liu L, He C. Cold acclimation by theCBF– COR pathway in a changing climate: lessons from *arabidopsis thaliana*. Plant Cell Rep. 2019;38(5):511–519. doi:10.1007/s00299-019-02376-3. 30652229 PMC6488690

[61] Lee HG, Seo PJ. The MYB 96– HHP module integrates cold and abscisic acid signaling to activate the CBF – COR pathway in Arabidopsis. The Plant J. 2015;82(6):962–977. doi:10.1111/tpj.12866. 25912720

[62] Yin S, Han Y, Huang L, Hong Y, Zhang G. Overexpression of HvCBF7 and HvCBF9 changes salt and drought tolerance in Arabidopsis. Plant Growth Regul. 2018;85(2):281–292. doi:10.1007/s10725-018-0394-4.

[63] Wisniewski M, Norelli J, Bassett C, Artlip T, Macarisin D. Ectopic expression of a novel peach (*Prunus persica*) CBF transcription factor in apple (Malus × domestica) results in short-day induced dormancy and increased cold hardiness. Planta. 2011;233(5):971–983. doi:10.1007/s00425-011-1358-3. 21274560

[64] Dou H, Xv K, Meng Q, LI G, Yang X. Potato plants ectopically expressing A rabidopsis thaliana CBF 3 exhibit enhanced tolerance to high-temperature stress. Plant, Cell & Environment. 2015;38(1):61–72. doi:10.1111/pce.12366. 24811248

