# Supplementary material for: Genome-wide identification and expression analysis of the CBF transcription factor family in Lolium perenne under abiotic stress
Source: Plant Signal Behav. 2022 Jun 17;18(1):2086733. doi: 10.1080/15592324.2022.2086733 (PMC10730156; doi:10.1080/15592324.2022.2086733)
Supplement: Supplemental Material [file KPSB_A_2086733_SM8652.docx]

| eIF4A-qPCR-F | AACTCAACTTGAAGTGTTGGAGTG |
| --- | --- |
| eIF4A-qPCR-R | AGATCTGGTCCTGGAAAGAATATG |
| LpCBF3a-qPCR-F | TGACGGAGGAAGAGGAGGAG |
| LpCBF3a-qPCR-R | CGATTCCTGCCTCAACTTCG |
| LpCBF3b-qPCR-F | CGAAGTTGAGGCACACGTC |
| LpCBF3b-qPCR-R | GACAAAGTTCAGGGAGACGC |
| LpCBF3c-qPCR-F | TGACGGAGGAAGAGGAGGAG |
| LpCBF3c-qPCR-R | CGATTCCTGCCTCAACTTCG |
| LpCBFVb-qPCR-F | CGAGCTGATGACGACCTTCT |
| LpCBFVb-qPCR-R | CCTCAACTTCGCCGACTCC |
| LpCBFIVb-qPCR-F | GATGAGTCGGCGAAGTTGAG |
| LpCBFIVb-qPCR-R | GGCGGACAAAGTTCAAGGAA |
| LpCBFIVa-qPCR-F | GAACTCCTCGTCCCTTGCA |
| LpCBFIVa-qPCR-R | CGTCAGACCAGGAGAAGGG |
| LpCBFIIIc-qPCR-F | TCGTAATCACCGTTCTCCCA |
| LpCBFIIIc-qPCR-R | AATGTTTCAGGTTCCGGCTG |
| LpCBFIIIb-qPCR-F | GATCGCCGTAATCACCGTTC |
| LpCBFIIIb-qPCR-R | GAATGTTTGAGGTTCCGGCA |
| LpCBFIIIa-qPCR-F | GCGAAGTTGAGGCACGAC |
| LpCBFIIIa-qPCR-R | CACCAAGTTCAAGGAGACGC |
| LpCBFII-qPCR-F | AGAGAAAGTCGGCACCTCAA |
| LpCBFII-qPCR-R | CCTCAACTTCCCGGACTCC |
| LpCBFIb-qPCR-F | AAGTTGAGGCATGTGGAGGA |
| LpCBFIb-qPCR-R | GACCAAGTTCAAGGAGACGC |
| LpCBFIa-qPCR-F | CTGTGAGGTAGGTGCCCAG |
| LpCBFIa-qPCR-R | CCCTTCCTTGTCGTCATCCT |

Table S1. Primers used in this study

Table S2. Predicted subcellular localization of LpCBF proteins

| Gene Name | WoLF PSORT Prediction |
| --- | --- |
| LpCBF3a | nucl: 10, mito: 3, cyto: 1 |
| LpCBF3b | nucl: 6, cyto: 3, mito: 3, chlo: 1, plas: 1 |
| LpCBF3c | nucl: 10, mito: 3, cyto: 1 |
| LpCBFVb | cyto: 4, mito: 2, extr: 2, vacu: 1 |
| LpCBFIVb | nucl: 10.5, nucl_plas: 6, cyto: 2, chlo: 1 |
| LpCBFIVa | nucl: 10, nucl_plas: 6.5, chlo: 2, cyto: 1 |
| LpCBFIIIc | nucl: 12, chlo: 1, mito: 1 |
| LpCBFIIIb | nucl: 11, mito: 2, chlo: 1 |
| LpCBFIIIa | nucl: 8, mito: 4, cyto: 2 |
| LpCBFII | chlo: 6, nucl: 3, cyto: 3, mito: 1, plas: 1 |
| LpCBFIb | nucl_ plas: 6, nucl: 5, plas: 5, cyto: 3, mito: 1 |
| LpCBFIa | nucl_ plas: 5, nucl: 4, plas: 4, chlo: 3, mito: 2, cyto: 1 |

cyto, cytoplasimic; cysk, cytoskeleton; vacu, vacuole; nucl, nuclus; golg, Golgi bodies; plas, plasma membrane; mito, mitochondria; chlo, choloplast; extr, exracellular; The numbers indicate. 14 Nearest Neighbors.

Table S3.Number of CBF family members in different plant species

| Organism name | Gene/Protein Name | Accession/ Locus ID | Protein length (aa) |
| --- | --- | --- | --- |
| Arabidopsis (Arabidopsis thaliana) | AtCBF1 | AT4G25490 | 213 |
|  | AtCBF2 | AT4G25470 | 216 |
|  | AtCBF3 | AT4G25480 | 216 |
|  | AtCBF4 | AT5G51990 | 224 |
| Ryegrass (Lolium perenne) | LpCBF3a | ABK32847 | 237 |
|  | LpCBF3b | AAX57275 | 223 |
|  | LpCBF3c | ABK32848 | 237 |
|  | LpCBFVb | BAF36846 | 210 |
|  | LpCBFIVb | BAF36844 | 224 |
|  | LpCBFIVa | BAF36843 | 223 |
|  | LpCBFIIIc | BAF36842 | 247 |
|  | LpCBFIIIb | BAF36841 | 252 |
|  | LpCBFIIIa | BAF36840 | 242 |
|  | LpCBFII | BAF36839 | 236 |
|  | LpCBFIb | BAF36838 | 242 |
|  | LpCBFIa | BAF36837 | 237 |
| Barly (Hordeum vulgare) | HvCBF1 | AAX23684 | 217 |
|  | HvCBF2 | AAM13419 | 221 |
|  | HvCBF3 | AAX23692 | 249 |
|  | HvCBF4 | AAX23695 | 225 |
|  | HvCBF5 | AAX23698 | 214 |
|  | HvCBF6 | AAX23701 | 244 |
|  | HvCBF7 | AAX23704 | 219 |
|  | HvCBF9 | AAX23707 | 291 |
|  | HvCBF11 | AAX23718 | 218 |
|  | HvCBF12 | ABA01491 | 244 |
|  | HvCBF13 | ABA01492 | 252 |
|  | HvCBF14 | ABA01493 | 214 |
| Rice (Oryza sative) | OsCBF1 | AY327040 | 214 |
|  | OsCBF2 | AY114110 | 219 |
|  | OsCBF3 | AY258283 | 219 |
|  | OsCBF4 | XM_467125 | 287 |
|  | OsCBF5 | AY345233 | 238 |
|  | OsCBF6 | AY345234 | 219 |
|  | OsCBF7 | AY345235 | 253 |
|  | OsCBF8 | XM_483621 | 236 |
|  | OsCBF9 | XM_483622 | 238 |
|  | OsCBF10 | AF494422 | 238 |
|  | OsCBF11 | AY166833 | 218 |
|  | OsCBF12 | AY319971 | 218 |
|  | OsCBF13 | AY785894 | 218 |
|  | OsCBF14 | AY785895 | 253 |
|  | OsCBF15 | AY785896 | 219 |
|  | OsCBF16 | AY785897 | 219 |
|  | OsCBF17 | AF300970 | 238 |
| Wheat (Triticum aestivum) | TaCBFIa-A11 | ABK55354 | 218 |
|  | TaCBFII-5.1 | ABK55355 | 225 |
|  | TaCBFII-5.2 | ABK55356 | 219 |
|  | TaCBFII-5.3 | ABK55357 | 228 |
|  | TaCBFIIIc-3.1 | ABK55361 | 235 |
|  | TaCBFIIIc-3.2 | ABK55362 | 246 |
|  | TaCBFIIIc-B10 | ABK55364 | 240 |
|  | TaCBFIIIc-D3 | ABK55363 | 245 |
|  | TaCBFIVd-D22 | ABK55390 | 275 |
|  | TaCBFIVd-B22 | ABK55389 | 290 |
|  | TaCBFIVd-A22 | ABK55388 | 275 |
|  | TaCBFIVd-D9 | ABK55387 | 269 |
|  | TaCBFIVd-B9 | ABK55386 | 269 |
|  | TaCBFIVd-9.1 | ABK55385 | 269 |
|  | TaCBFIVd-B4 | ABK55384 | 222 |
|  | TaCBFIVd-4.1 | ABK55383 | 222 |
| Rye (Secale cereale ) | ScCBFIa-11 | ABY59777 | 204 |
|  | ScCBFII-5 | ABY59778 | 213 |
|  | ScCBFIIIa-6 | ABY59779 | 229 |
|  | ScCBFIIIc-10 | ABY59780 | 221 |
|  | ScCBFIIIc-3A | ABY59781 | 228 |
|  | ScCBFIIIc-3B | ABY59782 | 231 |
|  | ScCBFIIId-12 | ABY59783 | 232 |
|  | ScCBFIVa-2A | ABY59786 | 193 |
|  | ScCBFIVa-2B | ABY59787 | 193 |

Note: Arabidopsis Protein sequence obtained from arabidopsis genome database(<https://www.arabidopsis.org/>); Protein sequences of other species obtained from NCBI genome database(https://www.ncbi.nlm.nih.gov/).

|  |  | Positive (%) | | | | | | | | |  |  |  |
| --- | --- | --- | --- | --- | --- | --- | --- | --- | --- | --- | --- | --- | --- |
|  |  | LpCBF3b | LpCBF3a | LpCBF3c | LpCBFIa | LpCBFIb | LpCBFII | LpCBFIIIa | LpCBFIIIc | LpCBFIIIb | LpCBFIVa | LpCBFIVb | LpCBFVb |
| Identity  (%) | LpCBF3b | / | 84 | 84 | 59 | 58 | 54 | 57 | 59 | 57 | 58 | 56 | 54 |
|  | LpCBF3a | 79 | / | 99 | 62 | 59 | 57 | 61 | 62 | 62 | 55 | 62 | 56 |
|  | LpCBF3c | 78 | 99 | / | 62 | 59 | 58 | 61 | 62 | 62 | 56 | 61 | 56 |
|  | LpCBFIa | 50 | 49 | 49 | / | 90 | 74 | 79 | 74 | 75 | 57 | 54 | 53 |
|  | LpCBFIb | 46 | 49 | 49 | 87 | / | 74 | 79 | 74 | 76 | 56 | 55 | 54 |
|  | LpCBFII | 46 | 49 | 50 | 66 | 66 | / | 69 | 67 | 67 | 52 | 53 | 50 |
|  | LpCBFIIIa | 47 | 51 | 51 | 71 | 70 | 61 | / | 88 | 90 | 53 | 53 | 50 |
|  | LpCBFIIIc | 49 | 49 | 49 | 66 | 67 | 59 | 84 | / | 92 | 54 | 54 | 51 |
|  | LpCBFIIIb | 49 | 50 | 50 | 69 | 69 | 60 | 87 | 91 | / | 52 | 57 | 49 |
|  | LpCBFIVa | 47 | 45 | 46 | 46 | 48 | 47 | 44 | 46 | 44 | / | 92 | 75 |
|  | LpCBFIVb | 47 | 48 | 47 | 46 | 45 | 46 | 44 | 46 | 46 | 88 | / | 74 |
|  | LpCBFVb | 47 | 46 | 46 | 45 | 45 | 42 | 43 | 46 | 43 | 70 | 69 | / |

Table S4.Comparison of amino acid homologies among LpCBF protein sequences

| Gene Name | Template | sequence Identity  (%) | GMQE | QMEAN |
| --- | --- | --- | --- | --- |
| LpCBF3b | 2gcc.1.A | 42.86 | 0.17 | -2.45 |
| LpCBF3a | 3gcc.1.A | 42.86 | 0.16 | -3.04 |
| LpCBF3c | 3gcc.1.A | 42.86 | 0.16 | -3.03 |
| LpCBFIa | 3gcc.1.A | 46.77 | 0.16 | -1.90 |
| LpCBFIb | 3gcc.1.A | 46.77 | 0.14 | -1.42 |
| LpCBFII | 1gcc.1.C | 50.82. | 0.15 | -1.27 |
| LpCBFIIIa | 3gcc.1.A | 50.00 | 0.15 | -1.23 |
| LpCBFIIIc | 2gcc.1.A | 51.61 | 0.16 | -1.78 |
| LpCBFIIIb | 1gcc.1.C | 52.46 | 0.15 | -0.53 |
| LpCBFIVa | 3gcc.1.A | 46.03 | 0.17 | -2.70 |
| LpCBFIVb | 3gcc.1.A | 46.03 | 0.17 | -2.78 |
| LpCBFVb | 3gcc.1.A | 47.62 | 0.18 | -3.21 |

Table S5. Predicted three-dimensional structures of LpCBF proteins

Table S6. Functional enrichment in the networks of LpCBF proteins

| Gene Name | cluster | | Description |
| --- | --- | --- | --- |
| LpCBF3a | CL:78955 | mixed, incl. [2Fe-2S] binding domain, and Molybdopterin-binding domain of aldehyde dehydrogenase | |
|  | CL:78953 | mixed, incl. FAD binding domain in molybdopterin dehydrogenase, and Molybdopterin-binding domain of aldehyde dehydrogenase | |
| LpCBFIa | CL:151063 | Microtubule-associated protein 70, and Probable zinc-ribbon domain | |
|  | CL:150921 | mostly uncharacterized, incl. Probable zinc-ribbon domain, and Microtubule-associated protein 70 | |
|  | CL:139019 | mixed, incl. Dehydrin, and Late embryogenesis abundant protein | |
|  | CL:150921 | mostly uncharacterized, incl. Probable zinc-ribbon domain, and Microtubule-associated protein 70 | |
| LpCBFIIIc | CL:151063 | Microtubule-associated protein 70, and Probable zinc-ribbon domain | |
|  | CL:139021 | mixed, incl. Late embryogenesis abundant protein, and Late embryogenesis abundant protein, LEA_1 subgroup | |
|  | CL:139019 | mixed, incl. Dehydrin, and Late embryogenesis abundant protein | |
|  | CL:150921 | mostly uncharacterized, incl. Probable zinc-ribbon domain, and Microtubule-associated protein 70 | |
| LpCBFVb | CL:151063 | Microtubule-associated protein 70, and Probable zinc-ribbon domain | |
|  | CL:28984 | mixed, incl. Ethylene insensitive 3, and Ethylene-insensitive protein 2 | |
|  | CL:28985 | mixed, incl. Ethylene insensitive 3, and Natural resistance-associated macrophage protein | |
|  | CL:150921 | mostly uncharacterized, incl. Probable zinc-ribbon domain, and Microtubule-associated protein 70 | |

Functional enrichments in the network originate from Local network cluster (STRING).

|  | 4 ℃ | | | | | 42 ℃ | | | | | Salt | | | | | PEG | | | | | ABA | | | | |
| --- | --- | --- | --- | --- | --- | --- | --- | --- | --- | --- | --- | --- | --- | --- | --- | --- | --- | --- | --- | --- | --- | --- | --- | --- | --- |
| Gene name | 0h | 3h | 6h | 12h | 24h | 0h | 3h | 6h | 12h | 24h | 0h | 3h | 6h | 12h | 24h | 0h | 3h | 6h | 12h | 24h | 0h | 3h | 6h | 12h | 24h |
| LpCBF3b | -0.0018 | 4.142285 | 4.967664 | 4.329683 | 2.593855 | -0.0018 | 2.463156 | 1.884139 | 2.989684 | 1.680753 | -0.0018 | 3.932035 | 4.556502 | 4.452081 | 4.368291 | -0.0018 | 1.699901 | 5.673551 | 5.503194 | 1.636216 | -0.00099 | 1.830518 | 3.694908 | 3.674535 | 1.984697 |
| LpCBF3a | -0.00022 | 0.842698 | 1.758979 | 2.578784 | 0.13113 | -0.00022 | 0.541076 | 1.665771 | 2.08344 | -0.3003 | -0.00022 | 1.601901 | 2.240753 | 2.843743 | 0.589094 | -0.00022 | 2.745368 | 4.170831 | 4.716166 | 1.86561 | -0.00022 | 1.892002 | 3.238576 | 4.040892 | 0.696271 |
| LpCBF3c | 0.005433 | 2.301005 | 1.888655 | 1.006809 | 0.545299 | 0.005433 | -0.60825 | -2.16264 | -1.50168 | -4.14347 | 0.005433 | 1.699996 | 1.059004 | 1.157403 | 1.689074 | 0.005433 | 1.364133 | -1.15611 | -0.94972 | -2.32263 | 0.005433 | 2.025302 | 1.332594 | 2.089253 | -1.33496 |
| LpCBFIa | 0.000872 | 4.467148 | 5.620865 | 4.797531 | 3.635393 | 0.000872 | 3.432125 | 3.683502 | 2.853573 | 3.129715 | 0.000872 | 0.958698 | 2.639731 | 3.732551 | 0.466543 | 0.000872 | 2.995477 | 3.612421 | 3.022288 | 2.720217 | -0.00183 | 2.589277 | 2.557395 | 3.306872 | 3.471428 |
| LpCBFIb | -0.00059 | 3.763876 | 4.911595 | 4.446732 | 4.075549 | -0.00059 | 0.744471 | 2.370348 | 2.361469 | 1.212848 | -0.00059 | 3.834158 | 4.16776 | 3.550239 | 1.111089 | -0.00585 | 1.946831 | 2.968366 | 2.589421 | 2.630579 | -0.00585 | 2.424655 | 3.104474 | 2.909355 | 0.112941 |
| LpCBFII | -0.00048 | 3.056197 | 3.824018 | 4.490656 | 3.879066 | -0.00228 | 0.605737 | 1.908804 | 2.386006 | 1.826717 | -0.00048 | 3.630874 | 3.983154 | 4.080474 | 1.1631 | -0.00341 | 0.94152 | 3.07511 | 2.474064 | 0.570694 | -0.00676 | 1.469875 | 1.734035 | 2.164091 | 1.990053 |
| LpCBFIIIa | -0.00119 | 1.610399 | 2.76599 | 4.563373 | 1.034735 | -0.0235 | 0.289916 | 1.760084 | 1.534878 | -1.32964 | -0.00119 | 0.545768 | 2.052619 | 1.530289 | 1.117182 | -0.00119 | 0.834637 | 1.310959 | 1.865393 | -0.08351 | -0.00183 | 2.589277 | 2.557395 | 3.306872 | 3.471428 |
| LpCBFIIIc | -0.0007 | 2.417846 | 3.615082 | 3.036833 | 1.664785 | -0.0007 | 1.202363 | 2.726092 | 3.011024 | 1.716678 | -0.0007 | 2.922749 | 3.636061 | 3.928484 | 3.371249 | 0.00311 | 1.430816 | 1.589206 | 4.149556 | 0.328185 | 0.00311 | 2.580269 | 3.235628 | 1.007787 | 1.82249 |
| LpCBFIIIb | 0.004287 | 1.023614 | 3.279652 | 1.770795 | 0.900853 | -0.00249 | 0.458925 | 2.081598 | 1.600323 | 1.665296 | 0.000376 | 0.60869 | 1.798281 | 1.552608 | 0.775718 | 0.000376 | 1.310275 | 2.58701 | 2.477632 | 1.583908 | 0.000376 | -1.99382 | -0.21463 | -0.97668 | -0.20952 |
| LpCBFIVa | 0.00246 | 0.81495 | 3.810952 | 2.856093 | 1.552764 | 0.000956 | 0.760677 | 2.531772 | 2.739013 | 0.284262 | -0.00429 | 1.621555 | 1.875205 | 2.571371 | 1.168267 | 0.001377 | 0.960642 | 3.047138 | 2.863726 | 2.997135 | 0.00246 | 0.787045 | 1.657819 | 1.365371 | -1.21012 |
| LpCBFIVb | 0.002408 | 2.323395 | 3.688371 | 2.870852 | 0.777644 | 0.002408 | -1.93715 | -1.32806 | -0.63303 | -1.38041 | 0.002408 | 0.980562 | 1.694774 | 4.191619 | 2.11793 | 0.002408 | -3.48912 | -2.21833 | -1.47138 | -2.13515 | 0.002408 | 0.302232 | -1.05399 | -0.71108 | -2.25009 |
| LpCBFVb | 0.001064 | 4.492316 | 4.96909 | 4.479779 | 1.78579 | 0.001064 | 0.954348 | 0.815052 | 2.196015 | -0.02785 | 0.001064 | 1.587066 | 3.466999 | 2.351811 | 3.18047 | 0.003451 | 0.73414 | 3.17651 | 2.982255 | 0.444176 | 0.001626 | 1.767128 | 2.057552 | 1.645762 | -0.26289 |

Table S7. The original data of heatmap.


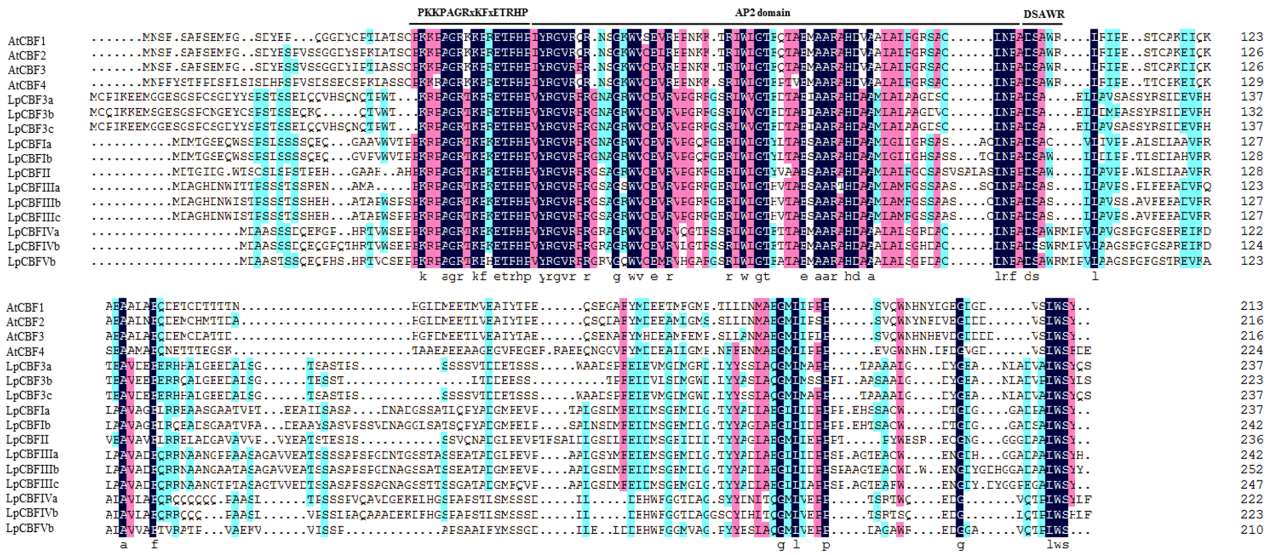
Figure S1. Multiple alignment of the amino acid sequences of LpCBF proteins.


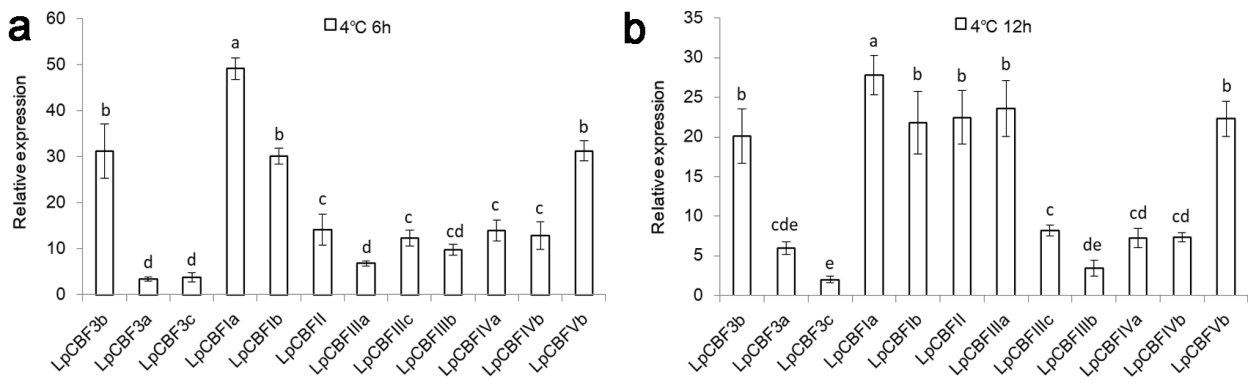


Figure S2. Expression of 12 *LpCBF* genes under 4°C for 6 h (a) and 12 h (b)
